# Context drives movement patterns in a mobile marine predator

**DOI:** 10.1186/s40462-023-00390-5

**Published:** 2023-05-24

**Authors:** Nicolas Lubitz, Ryan Daly, John D Filmalter, Marcus Sheaves, Paul D Cowley, Tor F Naesje, Adam Barnett

**Affiliations:** 1 Marine Data Technology Hub, College of Science and Engineering, James Cook University, Townsville City, QLD Australia; 2 Biopixel Oceans Foundation, Cairns, QLD Australia; 3 Oceanographic Research Institute, Marine Parade, PO Box 10712, 4056 Durban, South Africa; 4 South African Institute for Aquatic Biodiversity (SAIAB), Private Bag, 1015, 6140 Makhanda, South Africa; 5 Norwegian Institute for Nature Research, P.O. Box 5685, NO- 7485 Torgarden, Trondheim, Norway

**Keywords:** Animal movement, Context, Intra-specific variability, Resource availability, Bull sharks, Birds, Marine predators, Environmental change

## Abstract

Intra-specific variability in movement behaviour occurs in all major taxonomic groups. Despite its common occurrence and ecological consequences, individual variability is often overlooked. As a result, there is a persistent gap in knowledge about drivers of intra-specific variability in movement and its role in fulfilling life history requirements. We apply a context-focused approach to bull sharks (*Carcharhinus leucas*), a highly mobile marine predator, incorporating intra-specific variability to understand how variable movement patterns arise and how they might be altered under future change scenarios. Spatial analysis of sharks, acoustically tagged both at their distributional limit and the centre of distribution in southern Africa, was combined with spatial analysis of acoustically tagged teleost prey and remote-sensing of environmental variables. The objective was to test the hypothesis that varying resource availability and magnitude of seasonal environmental change in different locations interact to produce variable yet predictable movement behaviours across a species’ distribution. Sharks from both locations showed high seasonal overlap with predictable prey aggregations. Patterns were variable in the centre of distribution, where residency, small- and large-scale movements were all recorded. In contrast, all animals from the distributional limit performed ‘leap-frog migrations’, making long-distance migrations bypassing conspecifics in the centre of distribution. By combining multiple variables related to life history requirements for animals in different environments we identified combinations of key drivers that explain the occurrence of differing movement behaviours across different contexts and delineated the effects of environmental factors and prey dynamics on predator movement. Comparisons with other taxa show striking similarities in patterns of intra-specific variability across terrestrial and marine species, suggesting common drivers.

**Supplementary Information:**

The online version contains supplementary material available at 10.1186/s40462-023-00390-5.

## Background

Movement is a behavioural strategy by which mobile organisms fulfill life history requirements across time and space [1–3]. This includes acquiring energy by tracking food availability [4, 5], remaining in suitable environmental conditions, reproduction [6, 7] and avoiding predators, parasites or competition [3]. Animal movements, such as small-scale displacements and large-scale seasonal migrations, are important components of the life history strategies of many mobile species [1]. Such movements have consequences for ecological processes: They can lead to seasonal alteration in food web dynamics, facilitate connectivity between populations, and re-distribute nutrients/energy [2]. Therefore, improving knowledge on the drivers of animal movement is crucial to understand how environmental change and anthropogenic stressors interact with the ability to meet these requirements and ultimately affect ecological processes [8].

Despite apparent commonality of movement as a strategy to fulfill life history requirements, animal movement is inherently complex, with high levels of intra-specific variability evident in both terrestrial and aquatic taxa [9]. For example, residents and migrants can occur within the same population [10–13]. Differences also occur in the spatial extent and timing of movements, as well as routes and destinations [9, 11, 13, 14].

The behaviour of resident and migratory individuals is driven by life history requirements. Yet, movement patterns differ, suggesting that the tendency and spatio-temporal extent of movement as a strategy to fulfill life history requirements can be context-dependent [15, 16]. This context dependence may arise from the interaction of biotic and abiotic factors with an individual’s ability to fulfill life history requirements across time and space, producing intra-specific variability in movement [16, 17].

Despite its ecological and evolutionary consequences, this variability is often overlooked, with movement patterns averaged to produce summary population patterns. Ignoring potentially important variability hinders the development of a clear understanding of how groups of individuals within a species interact differently with their environment [15].

Instead of averaging data into population patterns, a context-focused approach incorporates intra-specific variability to better understand how complex and variable movement patterns arise [16]. The approach aims to define contexts under which specific movement patterns occur, given a set of contextual factors. These factors relate to the environment, ecology and the individual itself, such as prey availability, competition, seasonal temperature change, habitat composition, rainfall and ocean currents, reproductive strategy, sex and genetic-make up [3, 16]. An example of this approach is studying conspecifics from two or more study systems subjected to different contexts, such as environmental contexts at different geographic locations, or different individual contexts such as in males and females. Differences in contextual factors can then be related to differences in movement patterns to tease apart potential drivers of individual movement decisions [16]. Using context to predict movement patterns can aid in understanding how movement patterns may change if the context were to change, such as under climate change scenarios. Under this framework, movement drivers and resulting patterns can be compared between different taxa, aquatic and terrestrial.

Inter-specific variability in movement of terrestrial taxa, in particular birds, has been studied in more detail compared to marine species, which are more cryptic, harder to sample and often provide less location fixes with higher error levels when tracked [10]. An increased focus on marine species is required to better understand the degree to which similar life history constraints act across aquatic and terrestrial realms.

The bull shark (*Carcharhinus leucas)* is a large, mobile marine predator that occurs globally in coastal tropical, subtropical and warm-temperate zones [18]. Bull sharks also occupy freshwater and brackish habitats, where estuaries function as nurseries [19]. This wide distribution across a variety of climate/geographical zones and habitats makes them a suitable species to highlight the importance of context dependence in animal movement.

Bull sharks exhibit high variability in movement behaviour between and within geographical regions [20, 21], suggesting that movement behaviour is indeed context dependent. Drivers of movement are speculated to relate to a variety of factors. Including localised, seasonal food availability, such as provided by teleosts aggregating for spawning [21, 22] that can make up a significant proportion of bull shark diets [23]. Seasonal environmental change is also likely a driver as bull sharks migrate out of temperate regions (e.g. Sydney Harbour in Australia), when temperatures drop below a potential thermal limit of 19 °C for extended periods [24, 25]. Additionally, reproduction may drive movements, as females migrate into estuaries to pup [26]. Despite these proposed drivers, an empirical understanding of the specific contextual factors that interact to shape complex and variable movement patterns is missing [21].

As movement patterns and space use in large marine predators, including bull sharks, are expected to shift due climate change and other anthropogenic factors [27–29], we apply a context-focused approach to bull sharks, (as outlined in [16]), to test the hypothesis that differences in resource availability and magnitude of seasonal environmental change in different locations interact to produce variable yet predictable patterns of movement across a species’ distribution. Specifically, the aim was to determine how environmental factors and the occurrence of potential prey at bull shark aggregation sites drives movement strategies of animals tagged in two distinct geographic regions in southern Africa. Finally, we compare our results with terrestrial taxa to draw conclusions about the commonality of life-history constraints that shape movement patterns across aquatic and terrestrial realms.

## Methods

### Study site and acoustic array

**Fig. 1:**
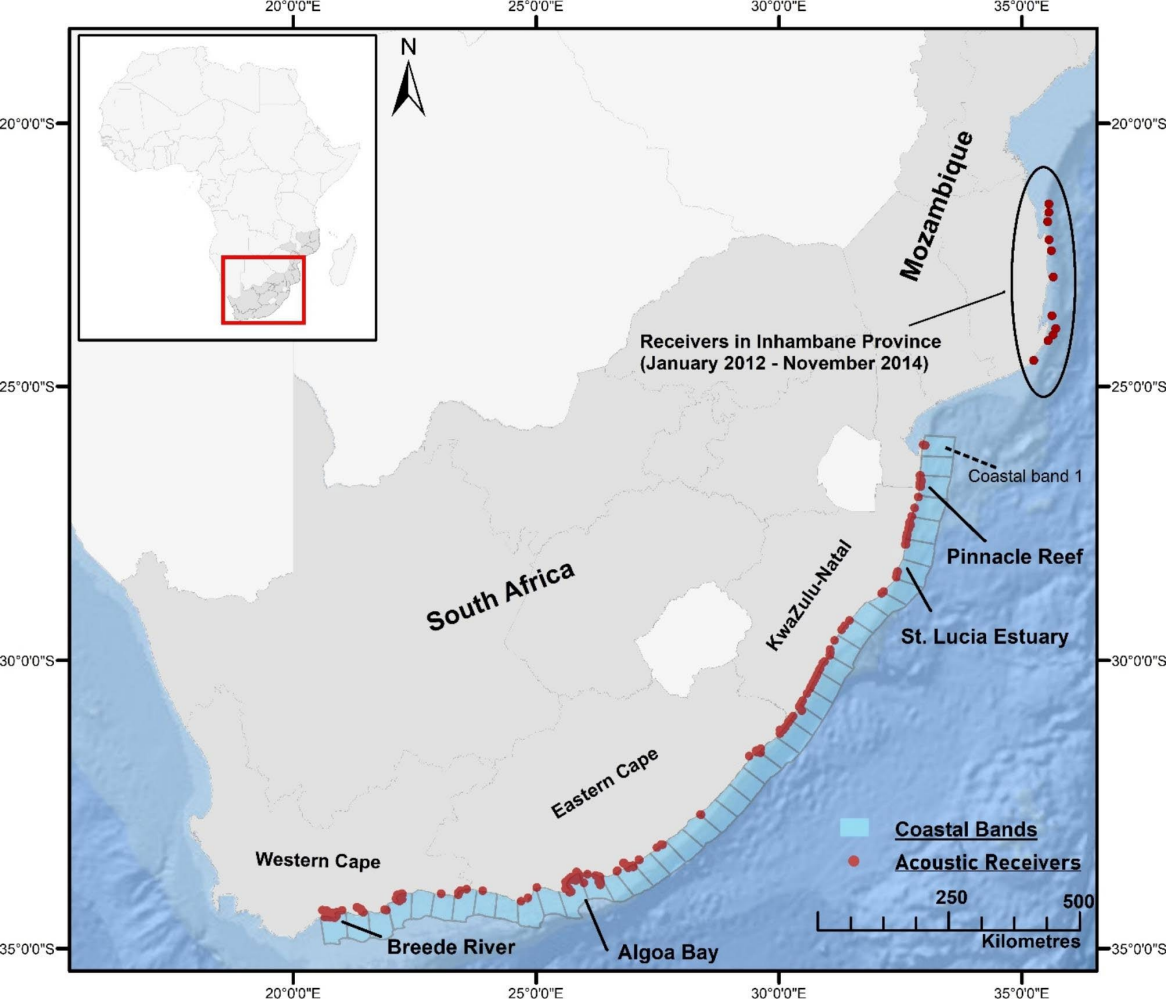
Map of the study area showing the extent of the acoustic array and coastal bands used for boosted regression tree modelling

The study was undertaken along the coast of southern Africa where the Acoustic Tracking Array Platform (ATAP) deployed an array of 158 InnovaSea VR2W acoustic receivers (Innovasea Ltd, USA) between January 2012 and March 2021 to track movements of bull sharks, and two coastal fish species that bull sharks may prey on, the giant trevally (*Caranx ignobilis*) and dusky kob (*Argyrosomus japonicus*) [23]. The array extends from west of the Breede River estuary (on the Western Cape of South Africa (34.48°S)) to northern Inhambane in Mozambique (21.54°S), spanning ca. 2000 km in latitudinal distance [30]. Receivers in Inhambane were only deployed from January 2012 until November 2014 with the most northern receivers deployed at Bazaruto Island and were thus not included in long-term modelling (see below). No receivers were deployed at Pinnacle Reef from May 2013 until May 2014, July 2014 until September 2014 and May 2015 until November 2015 (Fig. 1).

To investigate shark movement along the coast while accounting for changing receiver numbers during the monitoring period, the study area was divided into sections of coastline 40 km in length resulting in 41 coastal bands, excluding receivers deployed in northern Mozambique (Fig. 1). The size of coastal sections was chosen to ensure a maximum number of coastal bands with constant receiver deployment. Additionally, this length minimised spatial gaps in remotely sensed environmental variables while representing a meaningful spatial scale to capture changes in weekly space use of sharks.

The study area encompasses tropical, subtropical and warm temperate zones along the coast of southern Africa [31, 32]. Biophysical processes within most of the study region are dominated by the Agulhas Current, a strong western boundary current running along the narrow continental shelf gradually flowing south-west where it reaches the southern tip of a then widening continental shelf [31–33]. Wind and current driven upwelling systems are prominent features from the KwaZulu-Natal region to the Western Cape [34].

### Animal tagging and acoustic tracking

Between January 2012 and February 2020, 41 bull sharks were caught via rod and reel or set lines and implanted with high-powered InnovaSea V16 coded acoustic transmitters at two sites (battery life 1095–3652 days). Of those, 36 sharks were tagged at Pinnacle Reef, a subtropical reef complex located 3.5 km offshore within the marine component of the Maputo National Park (MNP). We considered this subtropical region as the centre of distribution for bull sharks. Additionally, five sharks were tagged in the estuary of the temperate Breede River, in the Western Cape of South Africa, at the distributional limit in southern Africa (supplemental material). These five sharks were also equipped with external PSAT-tags (miniPAT) which record depth, temperature and light-level for location estimates, which were pre-programmed to release from the animal after 180 days and float to the surface to transmit data. Based on differences in tagging location and space-use, bull sharks were separated into the Pinnacle Reef bull sharks and Breede River bull sharks for further analysis.

Sharks were monitored between January 2012 and March 2021. See [20] for details on tag specifications, capture and tagging methods. Between February 2015 and January 2020, 30 giant trevally (*Caranx ignobilis)* were caught via rod and reel and implanted with InnovaSea acoustic tags (V13 (n = 5) and V16 (n = 25)), (for full details of capture and tagging methodology for giant trevally see [35]). Additionally, 45 dusky kob (*Argyrosomus japonicus)* were caught via rod and reel and implanted with V16 InnovaSea acoustic tags in the Breede estuary between November 2015 and January 2020 (supplemental material). This animal study was conducted under ethics permits 25/4/1/7/5_2016-06-08 and 2013_06 granted by the South African Institute for Aquatic Biodiversity (SAIAB).

Acoustic data were analysed in the statistical software R and filtered for false detections (R Core team 2021) [36]. Days monitored were defined as the number of days between tagging date and date of last detection. Based on this, a residency index was calculated for each shark tagged at Pinnacle Reef and the Breede River estuary, as the number of days detected at Pinnacle Reef and the Breede River estuary, respectively divided by days monitored. The maximum displacement distance within the acoustic array was calculated as the length of coastline between the two most distant receivers a shark was detected on. This was extended for the Breede River sharks using the pop-off locations of the PSAT-tags. Pop-off locations were determined as the location and date where a consistent depth of 0 m was recorded.

### Environmental data acquisition

Environmental data were extracted from open-access data sets via the ERDDAP web server using the R package rerddapxtracto [37] and constituted quality-controlled remotely sensed data (see supplemental material for more explanation). Daily sea surface temperature and anomaly data in °C was obtained from the jplMURSST41 dataset at 0.01° resolution. Due to data availability, 8-day composites at 0.04° resolution for chlorophyll-a and photosynthetically available radiation, the component of electromagnetic radiation that can be used for photosynthesis, were extracted from the erdMH1chla8day and erdMH1par08day data sets respectively. Data for atmospheric pressure, which is linked to large-scale climate phenomena like the El Niño-Southern Oscillation (ENSO), was extracted from the erdlasFnPres6_LonPM180 data set every six hours at 1° resolution. Daily wind speed, which can influence upwelling, offshore transport and turbulence was extracted in m/s from the nceiPH533sstd1day data set at resolution of 0.04°. Finally, 5-day composite current data, which can impact biophysical processes primary production, species recruitment and distribution was extracted from the jplOscar_LonPM180 data set a at 1/3° spatial resolution.

All environmental variables were extracted at receiver locations and spatially averaged within each 40 km coastal band. Additionally, daily and composite values were compiled into weekly averages to investigate changes in weekly abundance of bull sharks within each coastal band.

### Data analysis

#### Influence of environmental context on bull shark abundance and movements

Boosted regression trees were used to assess influence of environmental variables on the number of tagged sharks detected per week in each coastal band, spanning the entire acoustic array from November 2015 until December 2020.

Boosted regression trees are a stochastic process with strong predictive performance that can identify important explanatory variables [38]. This machine learning technique is based on decision trees designed to improve model performance by iteratively fitting numerous models. See [38] and supplemental material 1 for more detailed descriptions of the application of boosted regression trees in ecology. We used the gbm.step function in the dismo package [39]. This function automatically performs cross validation (CV) to determine the optimal number of trees. Different combinations of values of learning rate, tree complexity and bag fraction were tested and the model with the lowest deviance and no signs of overfitting was chosen as the final model. Weekly abundance of bull sharks was modelled using a Poisson distribution. As number of sharks tagged and number of receivers deployed per coastal band changed over the analysis period, the number of sharks tagged and number of receivers deployed per coastal band in each week was included as an offset in the model formula. All environmental variables described above were included in the model with the addition of “month”, to investigate a seasonal effect, and “coastal band” to investigate the importance of location on abundance of tagged sharks. For the final model the fitted values were plotted to visualise the effect of each variable on weekly bull shark abundance within the acoustic array (supplemental material 1).

#### Influence of relative detection frequency of giant trevally and dusky kob at aggregation sites on bull shark movements

Acoustic data for giant trevally from Pinnacle Reef was available from November 2015 until May 2018, covering three annual spawning aggregations [35]. We used acoustic data for dusky kob from February 2019 until March 2021, the same time frame for which acoustic data from bull sharks tagged in the Breede estuary was available. Based on up to 12 years of observations on aggregation dynamics of both trevally and kob [20, 35, 40, 41], such as arrival time, abundance and aggregation duration, we treated the relative detection frequency of tagged giant trevally and dusky kob as a proxy for overall abundance (% of tagged animals detected in a given week).

To inspect a potential relationship between shark relative detection frequency and the annual giant trevally/dusky kob spawning aggregations, weekly relative detection frequency of bull sharks, giant trevally and dusky kob, calculated as number of sharks/trevally/kob detected per week, divided by total number of sharks/trevally/kob tagged in a given week, was plotted against time. Additionally, temperature data from a logger deployed 12 km south of Pinnacle Reef, as well as from a logger inshore from the Breede estuary (Fig. 1), were plotted in relation to relative detection frequencies.

To gain further insights into whether bull shark movements to and from Pinnacle Reef, in the centre of distribution, were influenced by specific phases of the trevally aggregation, such as arrival of trevallies or peak trevally relative detection frequency, we divided the study period into four distinct phases. The time outside the trevally spawning aggregation, when trevally were absent, was classified as phase one. Phase two began during the week trevally were first detected at Pinnacle Reef and ended the week where at least 50% of tagged trevally were detected for the first time. Phase three, the peak of the spawning aggregation, began when 50% or more of tagged trevally were detected for the first time and ended after 50% or more were detected for the last time. Phase four, began after the week 50% or more of tagged trevally were detected for the last time and numbers continuously declined until there were no more detections.

For 20 bull sharks tagged at Pinnacle Reef with sufficient, multi-year data available during the trevally modelling period (November 2015 -May 2018), number of days each shark was detected at Pinnacle Reef in each phase were summed and divided by the total number of days each phase lasted, resulting in individual detection probabilities per phase. This was modelled via logistic regression with binomial error structure against the phase of trevally aggregation, year and sex to determine the influence of trevally abundance, inter-annual variation and bull shark sex on shark detection frequency using the R package glmmTMB [42]. Shark ID was included as a random factor to account for repeated measures of the same individuals. The inclusion of explanatory variables and interactions as well as fitting of models with random intercepts and random intercepts and slopes was assessed using the Akaike Information Criterion (AICc) for small sample sizes. Fit of the selected model was then evaluated with diagnostics provided in the DHARMa package.

## Results

### Acoustic tracking and influence of environmental variables on bull shark abundance

#### Bull sharks tagged at pinnacle reef (centre of distribution)

**Fig. 2:**
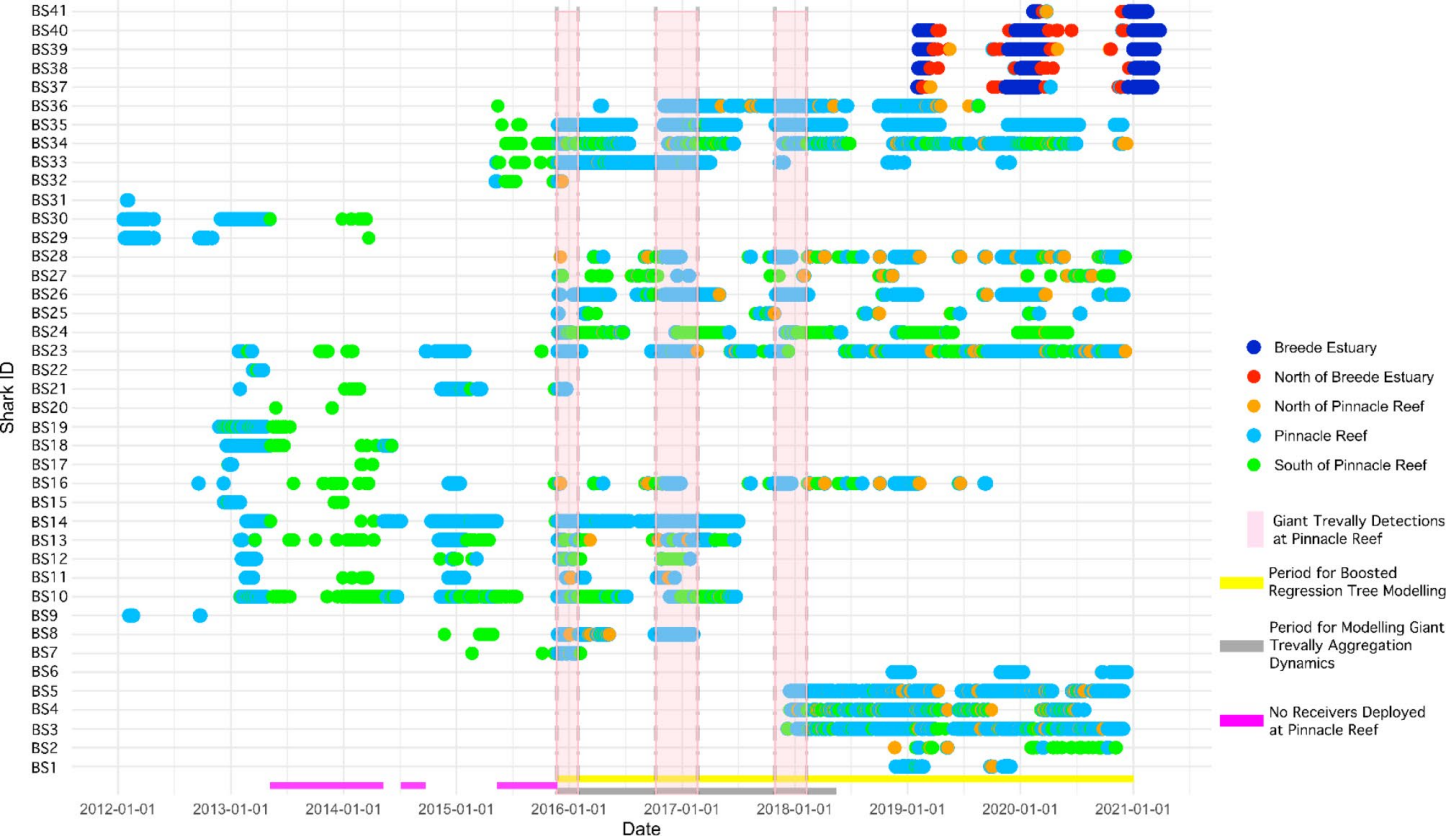
Timeline showing detections for 41 tagged bull sharks. For bull sharks BS37-BS41, tagged in the Breede River estuary, dark blue denotes detections in the Breede River estuary while red denotes any detections occuring north of the Breede River estuary but south of Pinnacle Reef. For bull sharks BS1-BS36, tagged at Pinnacle Reef, light green denotes any detections south of Pinnacle Reef. For all bull sharks, light blue denotes detections at Pinnacle Reef and orange denotes detections north of Pinnacle Reef

**Fig. 3:**
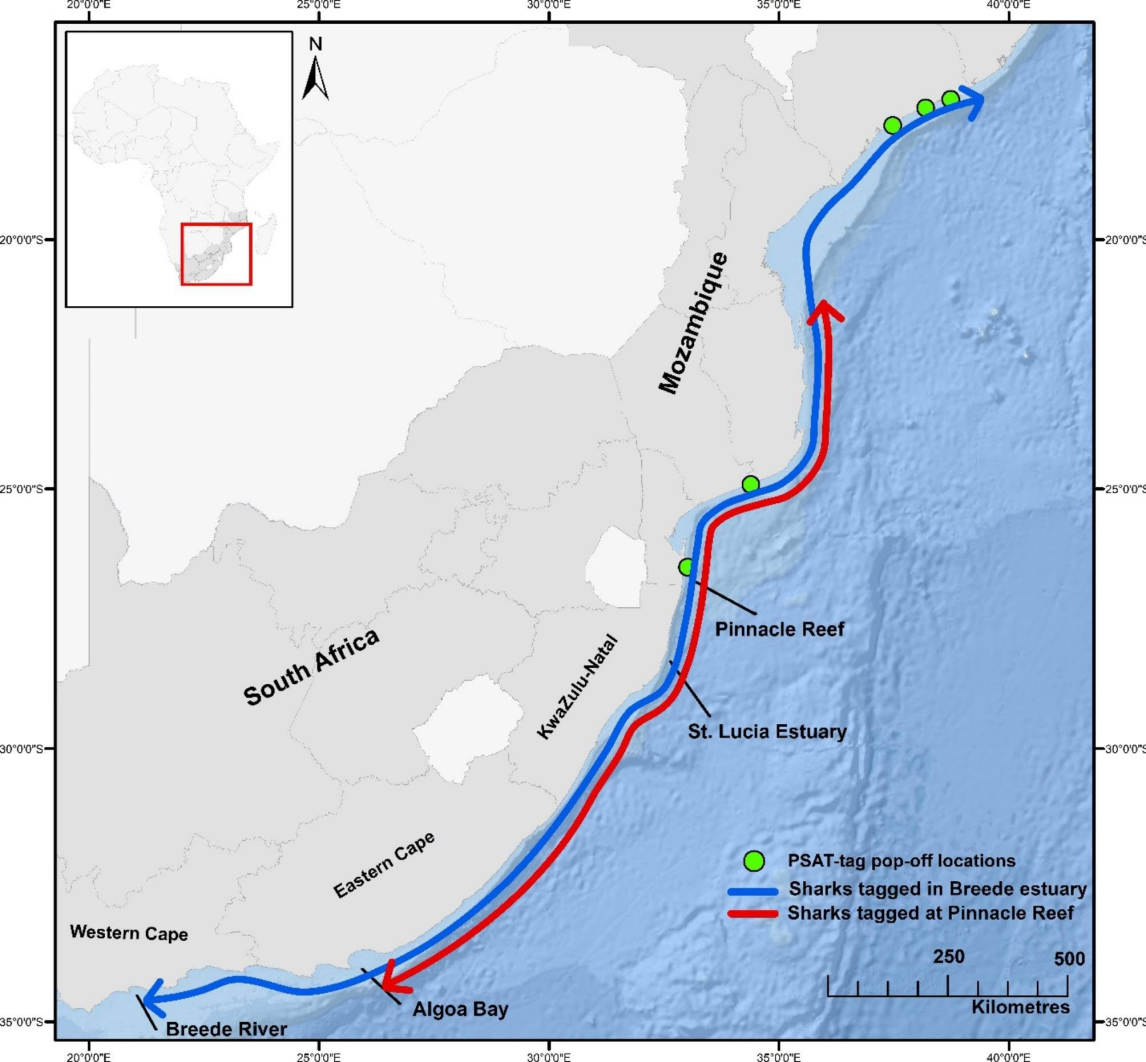
Extreme extents of bull shark movements along the coast of Southern Africa by sharks tagged at Pinnacle Reef in red and sharks tagged in the Breede River estuary in blue, demonstrating leap-frog migrations performed by Breede River sharks. Also shown are the PSAT-tag pop-off locations for bull sharks tagged in the Breede River estuary (n = 5)

Overall, sharks tagged at Pinnacle Reef (n = 36, males: 21, females 15) were monitored for up to 2875 days (range: 6-2875 days, mean: 1153) and had a mean residency index within the whole array of 0.19 (range: 0-0.6). All tagged individuals were likely mature (mean total length 253.6 cm) (Table 1) [23]. Pinnacle sharks were detected year-round on receivers from Inhambane province, Mozambique to Algoa Bay (spanning ca. 1800 km and 30 coastal bands, Figs. 1–2). However, some individuals were not detected in the array for weeks to months at a time (Fig. 2). Coastal band and month had the highest influence on weekly abundance of sharks tagged at Pinnacle Reef during the boosted regression tree modelling period from November 2015 until December 2020 (Table 2). Shark abundance was highest across years in coastal band three, which includes Pinnacle Reef. Additionally, between January 2012 and February 2020, 86.3% of detections of sharks tagged at Pinnacle Reef occurred at the tagging site, 11.7% occurred on receivers between Pinnacle Reef and the St. Lucia Estuary to the south, and the remaining 2% of detections occurred either to the south of the St. Lucia Estuary down to Algoa Bay or to the north of Pinnacle Reef along the coast of southern Mozambique (Figs. 1–3).

**Table 1:** Residency Index for bull sharks BS1-36 tagged at Pinnacle Reef and for BS37-41 tagged in the Breede River estuary. Maximum displacement is the maximum length of coastline between the two furthest receivers an individual was detected on. For BS37-41, tagged in the Breede River estuary, values in brackets indicate maximum displacement between the furthest receiver and the pop-off location for PSAT-tags.

SharkID	RI	Sex	Max. Displacement in km	SharkID	RI	Sex	Max. Displacement in km
BS1	0.24	F	85	BS22	0.09	M	12
BS2	0.006	M	724	BS23	0.27	F	873
BS3	0.48	M	161	BS24	0.03	M	273
BS4	0.28	F	206	BS25	0.02	M	1058
BS5	0.6	F	85	BS26	0.34	F	429
BS6	0.13	M	85	BS27	0.01	F	404
BS7	0.11		12	BS28	0.19	F	723
BS8	0.2	M	45	BS29	0.11	F	1326
BS9	0.05	F	463	BS30	0.22	M	12
BS10	0.1	M	131	BS31	0.21	M	473
BS11	0.09	F	625	BS32	0.07	F	23
BS12	0.05	F	1330	BS33	0.26	M	104
BS13	0.2	F	1836	BS34	0.16	M	195
BS14	0.47	M	496	BS35	0.43	M	45
BS15	0.09	M	12	BS36	0.35	F	130
BS16	0.09	F	1380	BS37	0.26	M	1683 (3024)
BS17	0.02	M	493	BS38	0.16	M	1610 (2518)
BS18	0.15	M	12	BS39	0.38	M	1683 (1760)
BS19	0.23	M	12	BS40	0.3	M	1610 (2995)
BS20	0	M	120	BS41	0.2	M	1622 (3154)
BS21	0.06	M	120				

**Fig. 4:**
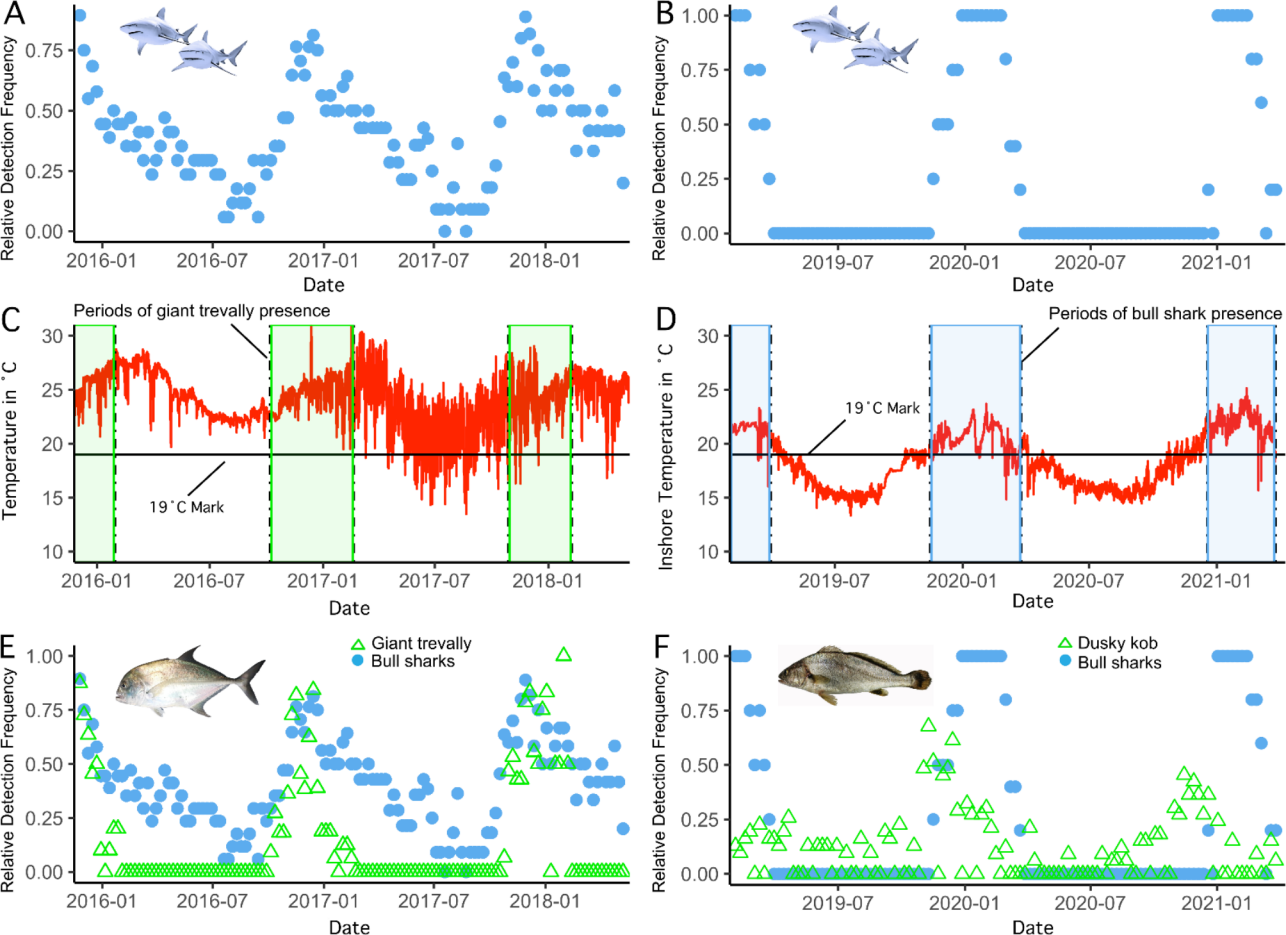
A) Relative detection frequency of bull sharks at Pinnacle Reef, B) relative detection frequency of bull sharks (*Carcharhinus leucas*) in the Breede estuary, C) temperature 10 km south of Pinnacle Reef, showing the 19 °C mark corresponding to near-zero bull shark abundance in Sydney Harbour, Australia [24] and trevally presence shaded in green, D) temperature from inshore, near the Breede River mouth, showing the 19 °C mark and bull shark presence shaded in blue, E) relative detection frequency of bull sharks and giant trevally (*Caranx ignobilis*) at Pinnacle Reef, F) relative detection frequency of bull sharks and dusky kob (*Argyrosomus japonicus*) in the Breede estuary

83.9% of detections occurred between October and April every year, with weekly shark abundance peaking in coastal band three from November until January. Sharks were detected year-round, with the lowest numbers detected during the Austral winter.

All environmental variables had negligible effects on weekly bull shark abundance within coastal bands, but sea surface temperature was responsible for most splits in the model for Pinnacle Reef sharks (0.9% relative influence, Table 2). These sharks were detected in an average weekly temperature range of 20.5˚C to 28.6˚C with highest abundance occurring between 24˚C and 27˚C (supplemental material). Data from the temperature logger deployed near Pinnacle Reef at 30 m depth suggests that sharks may experience incursions of cold water, sometimes as low as 13˚C during short-lasting, upwelling events (Fig. 4). However, sharks may evade this cold water by remaining above the thermocline (Ryan Daly, personal observation).

While receivers were deployed in Inhambane Province, Mozambique from January 2012 until November 2014, 10 sharks were detected there during the Austral winter, suggesting that some sharks migrate at least 600 km to the north of Pinnacle Reef (Fig. 1). On average females made longer movements within the array compared to males (mean = 661.2 km (range: 85- 1836 km), compared to 234.9 km (range: 12-1058 km), Table 1). Movements to the south of Pinnacle Reef occurred year-round but were most common during the Austral Summer while movements to the north of Pinnacle Reef were most common during the winter.

In general, bull sharks tagged at Pinnacle Reef showed high variability in movement with three general movement modes: (1) resident individuals with year-round occurrence at Pinnacle Reef and nearby receivers (n = 8, mean monitoring period: 1331 days) (Figs. 2 and 3), (2) individuals that were sporadically detected in the array without clear attendance patterns at Pinnacle Reef, and (n = 10, mean monitoring period: 693 days) (3) individuals that roamed widely in the array but returned to Pinnacle Reef between October and April (n = 18, mean monitoring period: 1351 days) (Figs. 2 and 3).

**Table 2:** Relative influence (in percentages) of each variable on abundance in all coastal bands of bull sharks tagged at Pinnacle Reef and the Breede River

Variable	Relative influence (Pinnacle sharks)	Relative influence (Breede River sharks)
Coastal band	83.55	28.52
Month	13.11	32.43
Sea surface temperature (˚C)	0.91	32.56
Sea surface temperature anomaly (˚C)	0.15	0.87
Wind speed (m/s)	0.44	1.14
Chlorophyll-a levels (mg/m^3^)	0.37	1.03
Photosynthetically available radiation (m^− 2d−1^)	0.37	0.38
Meridional current speed (m/s)	0.53	0.03
Zonal current speed (m/s)	0.58	3.04

#### Bull sharks tagged at the breede river estuary (distributional limit)

Bull sharks tagged at their distributional limit, in the Breede river (n = 5 mature males, mean total length: 260.5 cm) had a mean residency index of 0.26 (range: 0.16–0.38) in the entire acoustic array (Table 1). Sea surface temperature, month and coastal band had the highest influence on shark abundance during the boosted regression tree modelling period for this group of sharks, between February 2019 and December 2020 (Table 2). Highest abundance occurred in coastal band 41, which included the Breede estuary. Here, sharks remained during the austral summer and early autumn. During the overall monitoring period for sharks tagged at the Breede estuary, (February 2019 to March 2021) 99% of their detections occurred in and around the estuary, with the remaining 1% of detections occurring along the coast between the Breede estuary to ca. 70 km north of Pinnacle Reef (the most northern tip of the array after receivers in Inhambane were removed in November 2014). Thus, sharks migrated along the entire acoustic array, with PSAT-tag pop-off locations also demonstrating that these animals performed leap-frog migrations, migrating past conspecifics tagged at Pinnacle Reef, in the centre of distribution, covering ca. 3000 km of coastline one-way (Fig. 3).

Breede estuary bull sharks were detected within an average weekly temperature range of 17.8˚C to 27.5˚C, with highest abundance occurring between 20˚C and 22˚C (see supplemental material 1). These sharks were highly site attached to the Breede estuary, with daily up- and downstream movements evident in all individuals. All sharks tagged in the estuary left the Breede area, when temperatures exhibited a constant downward trend, usually below 19˚C in February/March (Fig. 4D). During their north-ward migrations, these sharks experienced temperatures as low as 12˚C for short periods of time between the Breede estuary and Algoa Bay. Acoustic detections ceased near Pinnacle Reef, usually between April and May (Figs. 1 and 3). The Breede River sharks were not detected again until October/November, when they were detected at or near Pinnacle Reef moving southward towards the Breede River (Figs. 1–3). Time spent at receivers outside the Breede estuary was short, with few detections lasting longer than 5 h. Seasonal movements between the Breede River and northern Mozambique occurred within 4–6 weeks, suggesting fast, directed migrations of at least 3000 km (mean = 2690.2 km, Table 1; Figs. 1–3). No periods of residency at Pinnacle Reef were evident by bull sharks tagged at the Breede estuary.

### Influence of giant trevally and dusky kob relative detection frequency on bull shark movements

At the Breede estuary, individual kob were often not detected for weeks at a time and likely left the estuary, but overall dusky kob were present year-round in the river with a peak in detections from November until January (Fig. 4) during the spawning season [43]. The bull sharks tagged at the Breede estuary were present from October until March, overlapping partially with the peak in dusky kob relative detection frequency (Fig. 4B, F). However, temperatures dropped below the proposed long-term thermal tolerance (19 °C) of bull sharks [24] for most of the year (Fig. 2D).

At Pinnacle Reef, giant trevally numbers typically increased sharply in October or November [35] and remained high until February/March. Then, giant trevally were again absent until the next annual spawning aggregation (Fig. 4E). For bull sharks tagged at Pinnacle Reef relative detection frequency at Pinnacle Reef followed a similar pattern, increasing sharply from October/November, matching the peak of trevally relative detection frequency. However, shark numbers decreased at a slower rate with some remaining after giant trevally have departed (Fig. 4A, E). Nonetheless, although the number of consecutive days spent at Pinnacle Reef decreased when giant trevally were absent, sharks were detected year-round regardless of temperature, which rarely dropped below 19 °C (Fig. 4D, [24]).

**Fig. 5:**
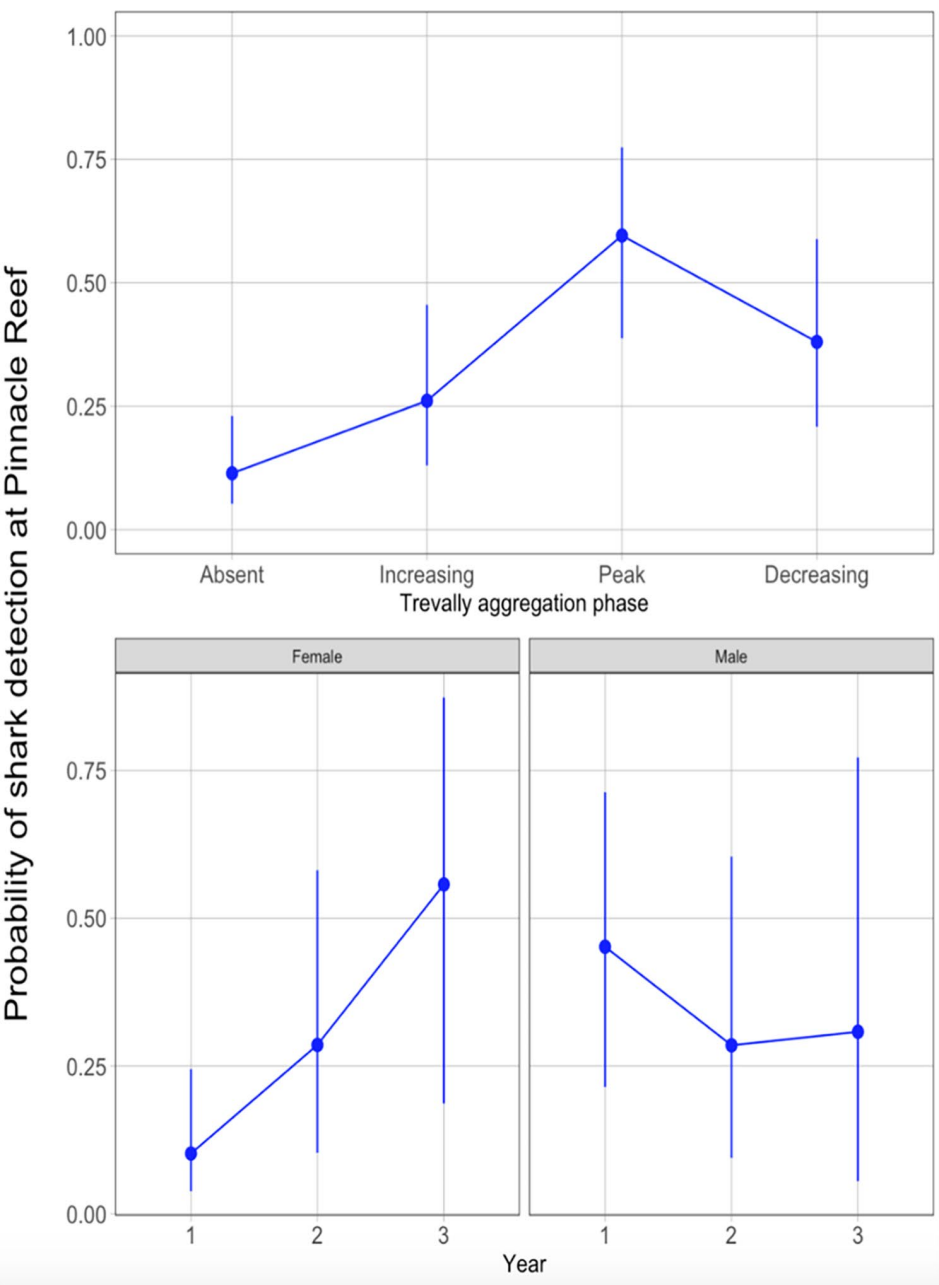
Logistic regression model results. Y-axis shows individual detection probability for bull sharks at Pinnacle Reef. In the upper panel, the x-axis represents trevally aggregation phase and in the lower panel it shows year

The random intercept and slope model with giant trevally aggregation phase and an interaction term between shark sex and year, had the best fit and lowest AICc. Bull sharks spent significantly less time at Pinnacle Reef when giant trevally were absent in comparison to when trevally numbers were increasing at the beginning of the spawning aggregation (p < 0.001), at the peak of the spawning aggregation (p < 0.001) and when giant trevally numbers are decreasing at the end of the aggregation (p < 0.001) (Fig. 5). However, the mixed model showed that variability between sharks was high (Fig. 5).

**Fig. 6:**
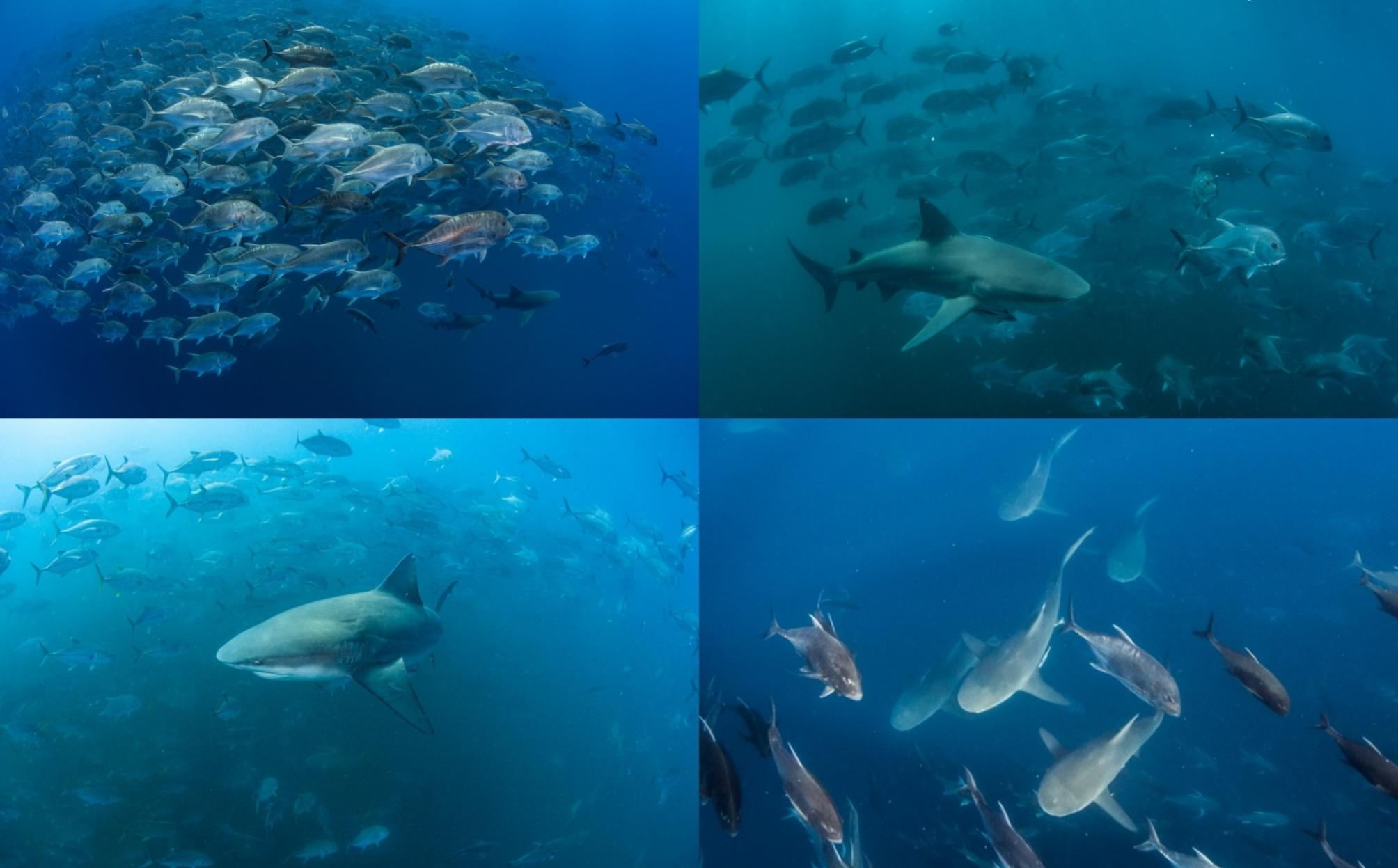
Bull sharks closely following giant trevally during the trevally spawning aggregation at Pinnacle Reef (Photo credit: Ryan Daly)

While some bull sharks showed strong association with the trevally aggregation, arriving on nearly the same day trevally were first detected and stayed for the whole spawning aggregation or longer, others were only detected occasionally or did not attend the aggregation in a given year (Figs. 5 and 6). Nonetheless, year did not have a significant effect on individual shark detection probability while sex did have a significant effect with females on average being detected less than males (Fig. 5). Finally, the sex- and year-interaction term revealed that females showed significant differences in their detection probability between aggregations while male detection probability did not change significantly over the three-year period (Fig. 5).

## Discussion

Intra-specific variation in movement behaviour appears to arise from intra-specific variation in the ability to fulfill life history requirements over time and space caused by an interaction of contextual environmental, ecological and individual factors [16, 44, 45]. In this study, a combination of sex, seasonal environmental change and timing and location of prey aggregations resulted in a variety of movement strategies in a mobile marine predator. This included residents with year-round detections, transients without clear temporal patterns, and migrants which showed site fidelity to focal points during times of increased prey availability. However, behavioural groups were not evenly distributed among locations. Animals tagged at the subtropical Pinnacle Reef showed high variability in movement behaviour, while sharks tagged at the distributional limit, in the Breede estuary, exhibited only large-scale return migrations interspersed with seasonal site fidelity. Despite a relatively small sample size for the Breede River sharks (n = 5 males), this uniform behaviour among individuals with limited variability tracked for three years provides confidence in the observed patterns. Additionally, as similar seasonality in abundance at bull shark distributional limits is observed in eastern Australia, including males and females, this is unlikely to be an artifact of male life history [26].

Regional differences in movement strategies, across varying contexts are becoming increasingly evident in terrestrial and marine taxa (e.g., [16, 46]. This is particularly well studied in birds. For example, broad-scale patterns of seasonal bird migration differ between flyways in Western and Eastern Europe based on spatio-temporal patterns of spring green-up and autumn senescence [47]. Similar to our observations, individuals which seasonally exploit areas at the range limit often make longer, less variable movements than individuals within the centre of distribution, which show higher variability [11, 28, 48–53].

Large seasonal fluctuations in environmental conditions and resource availability, such as at the distributional limit, place constraints on possible movement strategies, favouring repeated seasonality [54–57]. In populations of sea birds and marine turtles, this results in longer movements with increased foraging efforts, similar to the large-scale movements exhibited by the Breede estuary sharks which need to exploit a short window of suitable conditions and prey availability [50, 54, 55]. This is also evident in terrestrial taxa: Moose (*Alces alces*) are almost exclusively migrants in areas with more variable seasonal conditions producing higher snow depth and later snow melt [49], while African elephants (*Loxodonta Africana*) demonstrate the highest migration propensity in highly seasonal, arid environments [58].

Large magnitudes of seasonal temperature changes also impose direct physiological constraints: Pacific bluefin tuna (*Thunnus thynnus*), for example, seasonally leave productive feeding areas, migrating to warmer latitudes, in order to remain within thermal optima [59]. This demonstrates the importance of maximizing time spent within suitable temperatures over increased food availability, which is also a common trade-off for migratory bird species when tracking resources [60].

In southern Africa, teleosts make up a significant proportion of bull shark diets [23] and bull sharks tagged in the Breede River, an important spawning ground for dusky kob (*Argyrosomus japonicus)*, overlap with the kob spawning aggregation while seasonally present [43, 61]. However, shark presence showed a slight mismatch to peaks in prey abundance (Fig. 4). Hence, while migrations to this distributional limit appear driven by foraging opportunities, they need to be timed to match short windows of suitable temperatures (Fig. 4). Bull sharks seasonally leave Sydney Harbour, a productive estuary near the range limit in Australia, when temperatures consistently drop below a possible thermal limit of 19 °C (April/May) [24]. Similarly, in the Breede estuary, temperatures remain below 19 °C between March and October (autumn- spring) (Fig. 4).

At the distributional limit, seasonal migrations by bull sharks tagged at the Breede estuary are likely driven by strong seasonal temperature fluctuations (Fig. 4). Superficially, the same pattern is evident for sharks tagged at the subtropical Pinnacle Reef, in the centre of distribution, where shark abundance and detection frequency decrease during the cooler winter months (Fig. 4). However, winter temperatures at Pinnacle Reef remain generally higher than even the summer temperatures at the Breede River (Fig. 4), suggesting that seasonal temperature changes are an unlikely driver for movements of sharks tagged at the subtropical Pinnacle Reef. This demonstrates the benefit of comparing two geographically distinct groups, since when viewed in isolation seasonal change could mask the effects of other variables, such as fluctuations in prey availability (Fig. 4). Note, however, that only large-scale environmental change was investigated in the present study, but finer scale, local, environmental context may also influence marine predator movements.

Year-round suitable environmental conditions in the centre of distribution, such as at Pinnacle Reef, likely allow for diversification of movement strategies, where residents, transients and migrants can track resources without being constraint by temperatures. Model results show that peak abundance of bull sharks at Pinnacle Reef, including the arrival of non-resident sharks, matches the arrival of giant trevally (*Caranx ignobilis*) for their annual spawning aggregation, with some sharks returning the exact day giant trevally are first detected again (Figs. 4 and 5). This spawning aggregation represents the largest recorded for this species, comprising of at least 2413 individuals [41].

Despite differing environmental contexts among locations and resulting differences in movement strategies, site fidelity to exploit seasonal resource pulses occurs in both groups of tagged sharks. The Breede River sharks exhibit leapfrog migration, bypassing conspecifics in the centre of distribution, a common migration pattern in birds [62, 63]. For birds, leapfrog migration is a driver of intra-specific variation, believed to arise from differences in environmental context, resource availability and intra-specific competition [62].

The Breede sharks were detected at Pinnacle Reef during the trevally spawning aggregation, thus at the same time their conspecifics aggregate there to prey on spawning trevally. Yet, the Breede sharks did not exhibit any residency, migrating through this suitable feeding ground (Fig. 3). Similarly, sea turtles and birds show site fidelity to feeding grounds after returning from their breeding grounds, migrating past other suitable feeding habitat en route [64, 65]. Why individuals undergo pro-longed, costly migrations to disparate feeding habitats instead of remaining in foraging grounds that are closer remains unclear. However, across mobile terrestrial and aquatic taxa, individual risk mediation via site fidelity to predictable, seasonal prey aggregations and avoiding competition, could be vital life history strategies, especially when resources are patchily distributed in time and space [50, 64–69].

Reproductive behaviour can also lead to site fidelity and different life history requirements of males and females can shape differences in movement [52, 70–72]. For example, many terrestrial and marine species, exhibit natal philopatry [52, 72]. In this study, detection probability at the giant trevally aggregation at Pinnacle Reef remained stable for male bull sharks but fluctuated for females over the years (Fig. 5). As the trevally spawning aggregation occurs during the bull shark pupping season [23], females may not be able to fully attend this prey aggregation every year, due to the necessity to migrate to distant pupping grounds [21–26]. This may also explain why, of the sharks tagged at Pinnacle Reef, females, on average, showed higher migration distances than males (Table 1).

## Conclusion

Variability in movement behaviour is a wide-spread and important phenomenon in mobile species, adding substantial complexity [9–11]. In order to address this complexity and to demonstrate that incorporating intra-specific variability can improve our understanding of individual movement decisions, we applied a novel context-focused approach to bull sharks, incorporating variability and combining prey tracking with environmental variables [4, 16]. This macro-ecological approach has shown that variability in movement behaviour can be categorised into distinct behavioural groups, shaped by different contexts. Our results indicate that the composition of behavioural groups within a population is influenced by complex yet predictable interactions of differing environmental, ecological and individual contexts, such as differing magnitudes of environmental change, prey availability and sex differences.

When viewed in isolation and within a single geographical region, the effects of seasonal environmental change and prey availability on predator movements are often difficult to distinguish. Simultaneously investigating environmental conditions, and aspects of prey behaviour, predator movement and life history across different geographical regions allowed us to delineate the interplay of tracking prey availability, sex differences and environmental variables: In areas of large seasonal fluctuations in temperatures, seasonal large-scale movements are shaped by the necessity to remain in suitable temperatures, while exploiting short windows of prey availability. In contrast, smaller temperature fluctuations in the centre of distribution may mask the effect of seasonality in prey dynamics on predator movement, when in fact the lack of temperature constraints create variability in movement patterns.

Strong similarities in context-driven patterns of intra-specific variability and site fidelity occur across mobile aquatic and terrestrial species. This showcases the utility of incorporating intra-specific variability in order to explain movement outcomes based on a set of contextual factors and highlights the need for future studies to employ a macro-ecological, context-focused approach [15]. Especially, in the light of future climate change and resulting shifts in predictability to which long-distance migrants at the distributional limit are likely less adaptable (e.g., [53]), suggesting context-dependent effects of anthropogenic threats on different behavioural types.

## Electronic supplementary material

Below is the link to the electronic supplementary material.

Supplementary Material 1

## Data Availability

All acoustic tracking data for bull sharks, giant trevally and dusky kob are available from the Acoustic Tracking Array Platform at the South African Institute for Aquatic Biodiversity. All remote sensing data for environmental variables can be downloaded from the Erddap webserver (https://coastwatch.pfeg.noaa.gov/erddap/index.html).
