# Supplementary material for: Context drives movement patterns in a mobile marine predator
Source: Mov Ecol. 2023 May 24;11:28. doi: 10.1186/s40462-023-00390-5 (PMC10210390; doi:10.1186/s40462-023-00390-5)
Supplement: Supplementary file 1 — Supplementary Material 1 [file 40462_2023_390_MOESM1_ESM.docx]

**SUPPLEMENTAL MATERIAL**

**Tagging information for bull sharks implanted with acoustic transmitters (n=41), BS37-41 were also equipped with PSAT-tags**

| **Bull Sharks**  Shark ID Total Detections Tagging Date Last Detection Date Days Monitored Sex Total length in cm Tagging Location  BS1 4870 2018-11-23 2019-11-30 372 Female 263 Pinnacle Reef  BS2 1155 2018-11-20 2020-11-07 718 Male 220 Pinnacle Reef  BS3 25195 2017-12-08 2020-11-29 1087 Male 237 Pinnacle Reef  BS4 17842 2017-12-11 2020-07-26 958 Female 276 Pinnacle Reef  BS5 58528 2017-12-15 2020-11-29 1080 Female 285 Pinnacle Reef  BS6 4312 2017-12-15 2020-12-11 1093 Male 253 Pinnacle Reef  BS7 1940 2014-11-26 2016-02-07 439 Unknown Unknown Pinnacle Reef  BS8 6954 2014-11-21 2017-02-06 808 Male 241 Pinnacle Reef  BS9 108 2012-02-02 2012-09-24 235 Female 280 Pinnacle Reef  BS10 10981 2013-01-31 2017-06-23 1604 Male 283 Pinnacle Reef  BS11 5424 2013-02-18 2016-12-08 1389 Female 241 Pinnacle Reef  BS12 4038 2013-02-03 2017-01-27 1454 Female 261 Pinnacle Reef  BS13 12447 2013-01-30 2017-06-18 1600 Female 251 Pinnacle Reef  BS14 43679 2013-02-19 2017-07-02 1594 Male 251 Pinnacle Reef  BS15 1434 2012-12-09 2013-12-30 386 Male 244 Pinnacle Reef  BS16 19046 2012-09-18 2019-09-12 2551 Female 253 Pinnacle Reef  BS17 362 2012-12-20 2014-04-05 471 Male 264 Pinnacle Reef  BS18 1338 2012-12-18 2014-06-05 534 Male 263 Pinnacle Reef  BS19 2408 2012-11-23 2013-07-11 230 Male 282 Pinnacle Reef  BS20 51 2012-11-24 2013-11-25 367 Male 270 Pinnacle Reef  BS21 711 2013-01-26 2015-12-23 1062 Male 262 Pinnacle Reef  BS22 45 2013-01-29 2013-04-15 77 Male 195 Pinnacle Reef  BS23 27091 2013-01-24 2020-12-07 2874 Female 250 Pinnacle Reef  BS24 23244 2015-11-24 2020-06-03 1654 Male 266 Pinnacle Reef  BS25 1799 2015-11-25 2020-07-14 1693 Male 211 Pinnacle Reef  BS26 63309 2015-11-28 2020-11-29 1828 Female 254 Pinnacle Reef  BS27 3195 2015-11-28 2020-10-15 1783 Female 247 Pinnacle Reef  BS28 36267 2015-12-03 2020-12-06 1830 Female 265 Pinnacle Reef  BS29 4646 2012-01-23 2014-03-23 790 Female 220 Pinnacle Reef  BS30 4411 2012-01-19 2014-03-15 786 Male 250 Pinnacle Reef  BS31 13 2012-01-30 2012-02-03 5 Male 260 Pinnacle Reef  BS32 2943 2015-05-08 2015-12-09 215 Female 227 Pinnacle Reef  BS33 25112 2015-05-09 2019-11-28 1664 Male 286 Pinnacle Reef  BS34 27008 2015-05-12 2020-12-11 2041 Male 262 Pinnacle Reef  BS35 44358 2015-05-13 2020-11-26 2024 Male 241 Pinnacle Reef  BS36 52328 2015-05-14 2019-08-19 1558 Female 281 Pinnacle Reef  BS37 34927 2019-02-02 2021-03-03 682 Male 256 Breede River  BS38 9573 2019-02-05 2021-03-06 686 Male 286 Breede River  BS39 43422 2019-02-06 2021-03-10 623 Male 222 Breede River  BS40 26977 2019-02-07 2021-03-27 667 Male 278 Breede River  BS41 1247 2020-02-12 2021-02-15 323 Male 297 Breede River |
| --- |

2.

**Tagging information for dusky kob implanted with acoustic transmitters (n=45)**

| Length in cm | Sex | Tagging Date | ID |
| --- | --- | --- | --- |
| 128 | / | 15/11/2015 5:45 | 1 |
| 145 | FEMALE | 5/10/2016 9:46 | 2 |
| 121 | / | 18/10/2016 10:44 | 3 |
| 146 | / | 3/10/2016 8:24 | 4 |
| 127 | / | 19/10/2016 17:10 | 5 |
| 151 | / | 18/10/2016 15:15 | 6 |
| 114 | / | 20/10/2016 9:14 | 7 |
| 138 | FEMALE | 16/10/2016 18:40 | 8 |
| 123 | / | 19/10/2016 16:45 | 9 |
| 140 | FEMALE | 18/10/2016 13:56 | 10 |
| 114 | MALE | 29/10/2016 6:27 | 11 |
| 134 | / | 20/10/2016 4:28 | 12 |
| 121 | / | 29/10/2016 9:08 | 13 |
| 128 | / | 16/10/2016 5:14 | 14 |
| 129 | / | 19/10/2016 16:20 | 15 |
| 124 | MALE | 2/11/2016 9:12 | 16 |
| 119 | MALE | 2/11/2016 9:52 | 17 |
| 116 | MALE | 2/11/2016 10:02 | 18 |
| 123 | MALE | 20/10/2016 5:48 | 19 |
| 134 | FEMALE | 2/11/2016 12:10 | 20 |
| 140 | FEMALE | 17/10/2016 6:29 | 21 |
| 131 | / | 29/10/2016 7:03 | 22 |
| 139 | / | 18/10/2016 16:34 | 23 |
| 138 | MALE | 19/10/2016 9:23 | 24 |
| 121 | / | 17/10/2016 9:20 | 25 |
| 129 | / | 29/10/2016 7:34 | 26 |
| 146 | / | 18/10/2016 16:17 | 27 |
| 146 | FEMALE | 11/11/2016 11:54 | 28 |
| 160 | / | 15/10/2016 16:40 | 29 |
| 123 | MALE | 2/11/2016 10:12 | 30 |
| 114 | MALE | 19/10/2016 14:45 | 31 |
| 118 | / | 19/10/2016 8:15 | 32 |
| 172 | / | 12/04/2017 8:00 | 33 |
| 122 | MALE | 24/02/2017 4:58 | 34 |
| 119 | MALE | 2/11/2016 9:41 | 35 |
| 118 | MALE | 20/10/2016 5:08 | 36 |
| 149 | / | 29/10/2016 8:45 | 37 |
| 101.2 | MALE | 22/10/2018 13:30 | 38 |
| 104.3 | / | 16/12/2018 7:22 | 39 |
| 110 | MALE | 20/12/2018 17:27 | 40 |
| 109.8 | MALE | 22/01/2019 7:11 | 41 |
| 101.5 | MALE | 11/01/2020 14:43 | 42 |
| 129.4 | MALE | 12/01/2020 7:50 | 43 |
| 128.5 | / | 6/01/2019 11:07 | 44 |
| 115.6 | / | 19/12/2018 10:02 | 45 |

3.

**Summary of parameters and output values of final boosted regression tree models for sharks tagged at Pinnacle Reef and at the Breede River**

| **Location** | **Response variable** | **Number of trees** | **Learning rate** | **Tree complexity** | **Bag fraction** | **Mean total deviance** | **Residual total deviance** | **Estimated CV deviance (± SE)** | **CV (± SE)** |
| --- | --- | --- | --- | --- | --- | --- | --- | --- | --- |
| Pinnacle Reef | Weekly bull shark abundance per coastal band | 2050 | 0.01 | 4 | 0.5 | 2.228 | 0.395 | 0.453 (± 0.014) | 0.935 ± 0.003) |
| Breede River | Weekly bull shark abundance per coastal band | 1750 | 0.001 | 5 | 0.5 | 0.593 | 0.255 | 0.324 (± 0.032) | 0.771 (± 0.042) |

**More detailed description of application of boosted regression trees, also see Elith et al. 2008**

Boosted regression trees were used to assess the influence of the environmental variables on the weekly abundance of bull sharks in each coastal band from November 2015 until December 2020. This was the time period during which receivers in each coastal band were well integrated into the array with near-constant receiver deployment. Abundance was calculated as the sum of individual sharks present each week in each coastal band.

Boosted regression trees are a stochastic process that can reliably identify important explanatory variables and are characterised by strong predictive performance (Elith et al. 2008). In summary, boosted regression trees are a machine learning technique based on decision trees that intend to improve model performance by iteratively fitting numerous models. Regression trees are combined with “boosting”, a machine learning technique that adds trees step by step, aiming at reducing variability to reach the minimum level of deviance (see Elith et al. 2008 for a detailed description of the application of boosted regression trees in ecology).

Generally, a model with over 1000 fitted trees is more likely to achieve minimum deviance and maximum predictability and is thus preferred (Elith et al. 2008). In order to reach best model performance and minimum deviance the model parameters learning rate, tree complexity and bag fraction have to be chosen carefully to achieve a sufficient number of fitted trees. Elith et al. 2008 showed that a slower learning rate is preferable in order to reliably evaluate the response (Elith et al. 2008). As tree complexity increases fewer trees are generated for a given model, thus learning rate is decreased to fit a sufficient number of trees. In most instances a bag fraction between 0.5 and 0.7 is adequate (Elith et al. 2008). We used the gbm.step function in the dismo package (R core team 2021) to fit boosted regression trees to our data set. This function automatically performs cross validation (CV) to determine the optimal number of trees to fit to the final model. Different combinations of values of learning rate, tree complexity and bag fraction were tested and the model with the lowest deviance and no signs of overfitting was chosen as the final model. Weekly abundance of bull sharks was modelled using a Poisson distribution within the gbm.step function. As the number of sharks tagged and number of receivers deployed per coastal band changed over the analysis period, the number of sharks tagged and number of receivers deployed per coastal band in each week was additively included as an offset in the model formula. All environmental variables described above were included in the model with the addition of the variables “month”, to investigate a seasonal effect as well as “coastal band” to investigate the importance of location on abundance of tagged bull sharks. For the final model we plotted the fitted values to visualise the effect of each variable on weekly bull shark abundance within the acoustic array.

4.

**Fitted values of the boosted regression tree modelling weekly abundance in each coastal band for bull sharks tagged at a) Pinnacle Reef and b) the Breede River. Fitted values on the y-axis represent relative weekly bull shark abundance and x-axis represents each variable included in the machine learning model**

1.
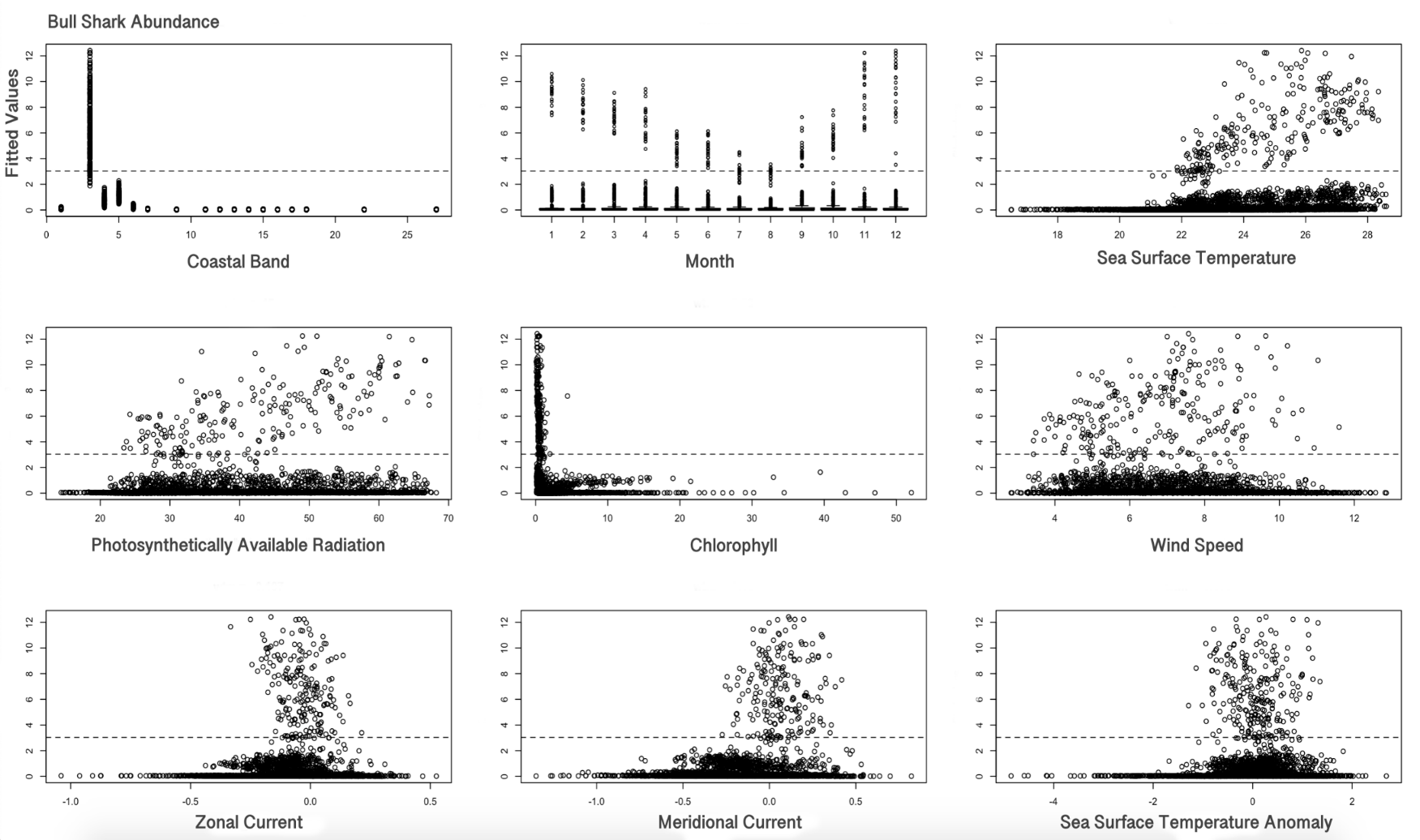


b)


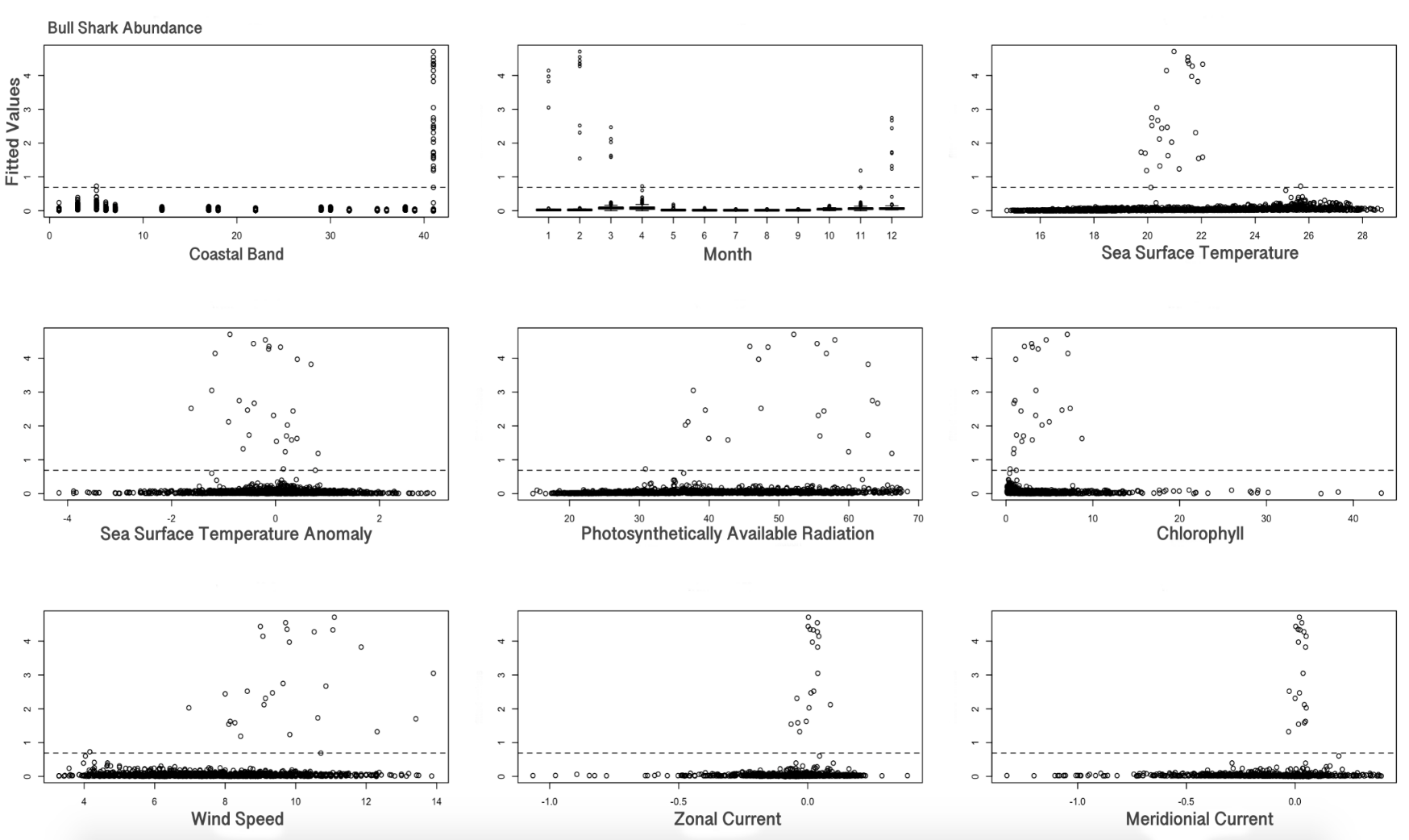


5.

**More detailed description of enviornmental variables**

Environmental data were extracted from open-access data sets via the ERDDAP web server using the R package rerddapxtracto in R and constitute processed, quality-controlled remotely sensed data. Daily sea surface temperature and sea surface temperature anomaly data in degree Celsius was obtained from the NASA Jet Propulsion Laboratory Multi-Scale Ultra-high Resolution Dataset (jplMURSST41) with a resolution of 0.01°.

Satellite data for proxies of productivity in the form of Chlorophyll-a and photosynthetically available radiation, the component of electromagnetic radiation that can be used for photosynthesis were extracted from the erdMH1chla8day and erdMH1par08day data sets respectively. Both data sets are collected from the NASA Aqua Spacecraft’s Moderate Resolution Imaging Spectroradiometer and are 8-day composites at 0.04° resolution.

Data for atmospheric pressure, which is linked to large-scale climate phenomena like the ENSO was extracted from the erdlasFnPres6_LonPM180 data set. This is gathered by the NOAA ERD and the FNMOC (Fleet Numerical Meteorology and Oceanographic Center) and provides pressure values every six hours at a resolution of 1°.

Wind speed, which can influence upwelling, off-shore transport and turbulence was extracted in m/s from the nceiPH533sstd1day data set created by the NOAA National Centers for Environmental Information. This data provides daily readings at a spatial resolution of 0.04°.

Finally, current data, which can impact biophysical processes such as upwelling and primary production, species recruitment and distribution was extracted from the jplOscar_LonPM180 data set gathered through the Ocean Surface Current Analyses Realtime (OSCAR) research project and constitutes a 5-day composite at 1/3° spatial resolution. This data set includes speed of the zonal current component in m/s which represents the current component that flows parallel to the equator and meridional current component in m/s which flows perpendicular to the equator.

All environmental variables were extracted at receiver locations and then spatially averaged within each 40 km coastal band. Additionally, daily and composite values were compiled into weekly averages to investigate changes in weekly abundance of bull sharks within each coastal band.

6.

**Model Output summary for the logistic model with lowest AICc investigating detection probability of bull sharks at Pinnacle Reef in relation to trevally aggregation phases**


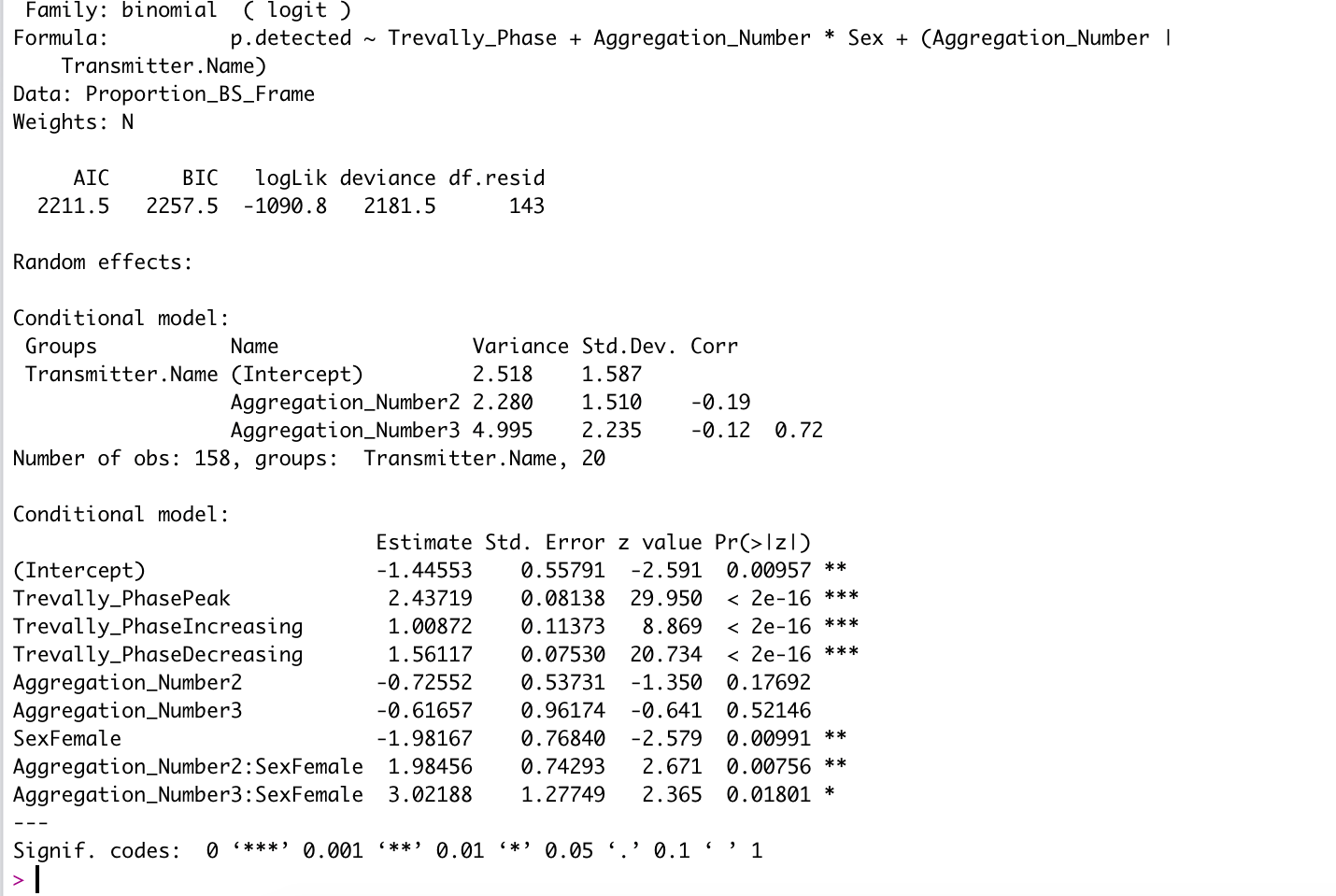


**References:**

Elith, J., J. R. Leathwick, and T. Hastie. 2008. A working guide to boosted regression trees. Journal of Animal Ecology **77**:802-813.
